# Exploring the causal relationship: bidirectional mendelian randomization study on benign prostatic hyperplasia and cardiovascular diseases

**DOI:** 10.3389/fgene.2024.1432055

**Published:** 2024-07-26

**Authors:** Nanyan Xiang, Shiqi Su, Zeng Wang, Yong Yang, Boxi Chen, Rui Shi, Tao Zheng, Banghua Liao, Yifei Lin, Jin Huang

**Affiliations:** ^1^ Department of Urology, Innovation Institute for Integration of Medicine and Engineering, Frontiers Science Center for Disease-Related Molecular Network, West China Hospital, Chengdu, Sichuan, China; ^2^ Engineering Research Center of Medical Information Technology, Ministry of Education, West China Hospital of Sichuan University, Chengdu, Sichuan, China; ^3^ Health Management Center, General Practice Medical Center, Innovation Institute for Integration of Medicine and Engineering, West China Hospital, Sichuan University, Chengdu, Sichuan, China; ^4^ West China School of Public Health, Sichuan University, Chengdu, Sichuan, China; ^5^ Department of Urology, West China Hospital, Chengdu, Sichuan, China; ^6^ Department of Urology, Innovation Institute for Integration of Medicine and Engineering, West China Hospital, Chengdu, Sichuan, China; ^7^ Program in Genetic Epidemiology and Statistical Genetics, Department of Epidemiology, Harvard T. H. Chan School of Public Health, Boston, MA, United States

**Keywords:** benign prostatic hyperplasia, cardiovascular diseases, mendelian randomization, genetic epidemiology, causal relationship

## Abstract

**Background:**

Benign prostatic hyperplasia (BPH) is a common disease occurring in elderly and middle-aged men, and cardiovascular diseases (CVDs) are one of the major causes of death worldwide. Many observational studies examined have found a strong association between BPH and CVDs, but the causal relationship between them is unclear. The aim of this study was to determine the causal relationship between BPH and CVDs, specifically five diseases: stroke, coronary heart disease (CHD), heart failure, myocardial infarction (MI), and atrial fibrillation (AF).

**Methods:**

In this study, we obtained single nucleotide polymorphisms (SNPs) of patients with BPH from the UK Biobank database and patients with CVDs from the UK Biobank, the HERMES Consortium, and the FinnGen Genome Database, each used as a genetic tool for a Mendelian randomization (MR) study. We used conventional MR analysis to assess potential causal direction between BPH and CVDs, as well as MR-Egger, MR-PRESSO, model-based estimation (MBE) and weighted median methods for sensitivity analysis.

**Results:**

Using a bidirectional two-sample MR study, we found that BPH patients had an increased risk of developing CHD (ConMix OR = 1.152, 95% CI: 1.011–1.235, *p* = 0.035) and MI (ConMix OR = 1.107.95% CI: 1.022–1.164, *p* = 0.013), but a decreased risk of stroke (ConMix OR = 0.872, 95% CI: 0.797–0.926, *p* = 0.002). The reverse study was not statistically significant and further research may be needed.

**Conclusion:**

Our study suggests a potential causal relationship between BPH and CVDs. BPH appears to be a risk factor for CHD and MI, but it may be protective against stroke. There was no evidence of a causal association in the reverse study, and a larger sample size was needed in follow-up to further explore the potential association.

## 1 Introduction

Benign prostate hyperplasia (BPH) is a prevalent chronic disease characterized by the enlargement of the prostate gland, frequently giving rise to lower urinary tract symptom (LUTS) (Dincer et al., 2021). This condition has evolved into a substantial public health concern, affecting over one million individuals globally in 20192). Notably, the incidence of BPH increased by 105.7% from 1990 to 2019. Its prevalence exhibits an age-dependent escalation, peaking at 14.67% among individuals aged over 70, with approximately 80% of men aged over 80 being affected (Zhang et al., 2019; Srinivasan and Wang, 2020; Zhu et al., 2021). Moreover, the economic impact of BPH was also substantial, evidenced by a significant rise in the expenses related to its diagnosis and treatment from 2014 to 2017. During this period, the average annual cost surged to $4 billion in the United States (Vuichoud and Loughlin, 2015; Del Giudice et al., 2022).

Although BPH is not life-threatening, it can present significant challenges marked by certain complications. Particularly within the context of prevalent age-related comorbidities such as cardiovascular diseases (CVDs), hypertension, diabetes, and the metabolic syndrome (Colon and Payne, 2008), certain individuals may experience moderate-to-severe lower urinary tract symptoms (LUTS) (McVary, 2006). Increasing researches have focused on the correlation between the presentation of BPH and the development of CVDs, including heart disease, stroke, and atrial fibrillation (AF) (Hägg et al., 2015; Davies et al., 2018; Sanderson et al., 2022; Wang et al., 2022). A prospective study of Chinese men demonstrated a significant association between BPH and cardiovascular diseases, heart disease, and stroke, particularly in men under 60 years of age (Burgess et al., 2013). Similarly, another study found a higher incidence of AF in individuals with BPH(10). Several observational studies have suggested a correlation between BPH and CVDs. However, due to the absence of randomized controlled trials (RCTs) or long-term cohort studies, the causal relationship between BPH and CVDs remains unclear.

Mendelian randomization (MR) is a method that uses genetic variation as an instrumental variable to determine causal relationships. This approach can effectively overcome bias caused by confounding and reverse causation, making it useful for causal inference studies (Daghlas et al., 2019). Several MR studies have investigated causal effects among BPH and CVDs. For example, depression has been shown to be causally associated with BPH and CVDs, including myocardial infarction (MI), AF, and coronary heart disease (CHD) (Wang et al., 2022). Using the two-sample MR framework, Yong-Bo Wang and Sara Hägg found that obesity and lifestyle factors also had a causal relationship between BPH and CVDs, such as heart failure and ischemic stroke (Hagg et al., 2015). However, no MR study has yet investigated the direct association between BPH and CVDs.

This study utilized a two-sample MR method (Davies et al., 2018) to investigate the causal relationship between BPH and CVDs, including stroke, CHD, heart failure, MI, and AF, with summary statistics from large-scale genome-wide association studies (GWAS), to help facilitate early screening for patients with BPH or CVDs.

## 2 Materials and methods

### 2.1 Research design and data sources

In our study, we employed a two-sample MR approach to investigate the causal relationship between BPH and five types of CVDs (stroke, CHD, heart failure, MI, and AF). We obtained BPH summary statistics from a meta-analysis of GWASs from the UK Biobank, while summary data for MI, stroke, and AF was also obtained from the UK Biobank, and heart failure and CHD was obtained from the HERMES Consortium and the FinnGen Genome Database, respectively. Further details on each dataset can be found in the Supplementary Table S1.

### 2.2 Selection the genetic variants associated with BPH and cardiovascular diseases

To ensure that genetic variants were strongly associated with the exposure under study, we selected genome-wide significant genetic variants associated with BPH and CVDs as instrumental variables for two-sample MR. All these genetic variants were clumped using PLINK (parameters: -clump-p1 5e-8 -- clump-p2 1e-5 --clump-r2 0.01 --clump-kb 500). Notably, no SNP was selected with the threshold of $p<5\times 10^{-8}$ for stroke, so we set p1 to 5e-6 to get adequate SNPs for analysis.

### 2.3 Mendelian randomization analysis

In this study, we employed various MR methods to examine the causal relationship between BPH and CVDs. To establish a causal effect through MR, three essential conditions must be satisfied: 1) genetic variants must be closely associated with the exposure under study, 2) the genetic variation must be independent of confounding factors, and 3) genetic variants must be associated with the outcome solely through the exposure, with no direct association due to horizontal pleiotropy (Sanderson et al., 2022).

The primary MR analysis was conducted using the inverse variance weighting (IVW) method (Burgess et al., 2013). If there was no horizontal pleiotropy, the results obtained through the IVW method remain unbiased. To address the potential impact of instrumental variable polymorphisms and extreme values, we also utilized the contaminated mixture method (ConMix) (Burgess et al., 2020) and Robust Adjusted Profile Score (RAPS) (Qingyuan et al., 2018), which could identify horizontal pleiotropy and acquire the proper results after removing outliers (Yang and Schooling, 2023). To ensure the validity of our results, we assessed the potential impact of horizontal pleiotropy on the instrumental variables using the MR-Egger test (Bowden et al., 2015). To address the issue of polymorphism, we used the PRESSO method to detect and remove outliers (Sun et al., 2023). Additionally, we used model-based estimation (MBE) and weighted median methods to calibrate our results.

Our analysis was conducted using R software version 4.2.1 and various packages, including "MendelianRandomization” for IVW, weighted median, MBE, and MR-Egger methods, "MR-PRESSO” for MR PRESSO, and "MR.RAPS” for building the RAPS model.

### 2.4 Ethics approval

This MR study was based on publicly available GWAS statistics, obviating the need for ethical approvals.

## 3 Results

### 3.1 The role of BPH in CVDs

To obtain the independent instrumental variable SNPs, we set the *p*-value threshold at $p<5\times 10^{-8}$ , and BPH was retained for CHD, stroke, heart failure, MI, and AF, with 28, 30, 29, 27 and 27 instrumental SNPs, respectively (Supplementary Table S2). Our bidirectional MR analysis provides evidence for a positive causal relationship between genetically predicted BPH and both CHD and MI, and a negative causal relationship between BPH and stroke. However, no significant causal relationships were found for heart failure and AF.

**FIGURE 1:**
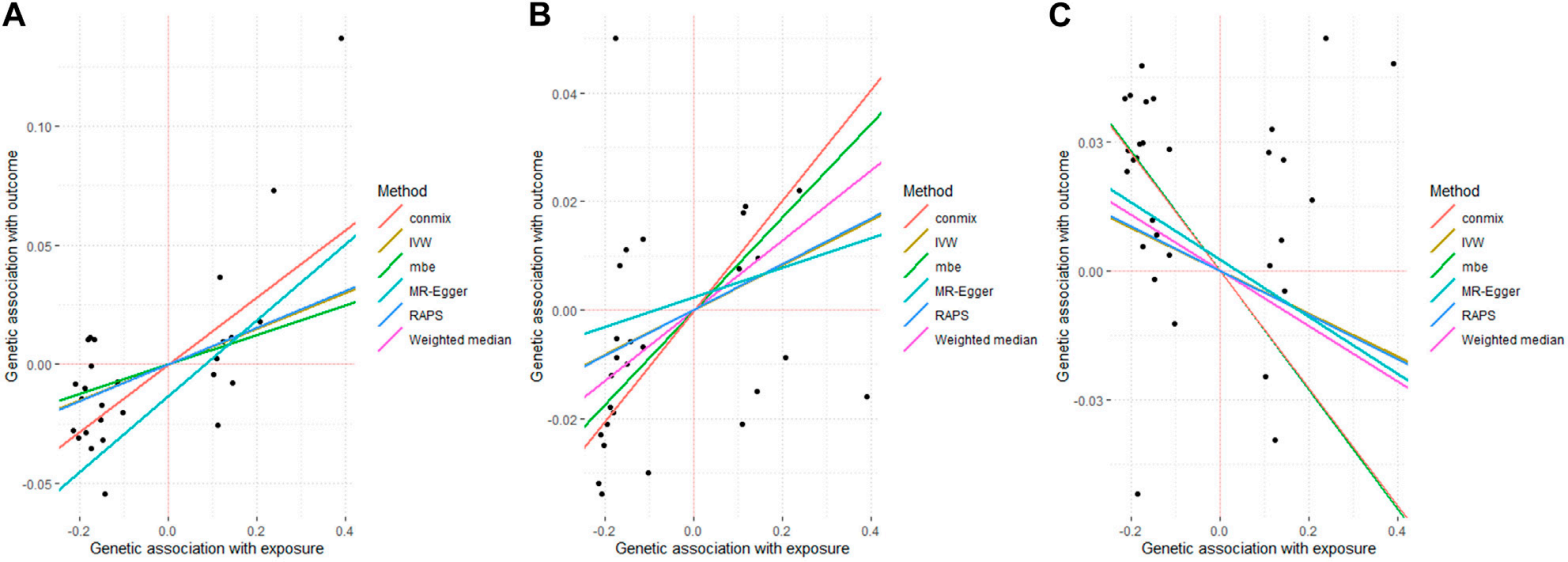
Associated plot of the main MR study investigating the causal effect of benign prostate hyperplasia on cardiovascular diseases. **(A)** outcome coronary heart disease **(B)** outcome myocardial infarction **(C)** outcome stroke.

First, genetically predicted BPH was found to be positively associated with CHD (ConMix OR = 1.152, 95% CI: 1.011–1.235, *p* = 0.035; RAPS OR = 1.08, 95% CI: 1.032–1.13, *p* = 0.001). The results of several other MR methods, including weighted median and IVW, were consistent with a causal effect of BPH on CHD (all OR > 1, *p* < 0.05) (Table 1; Figure 1). Moreover, the intercept of MR-Egger (*p* = 0.383) and the GlobalTest.*p*-value of MR-PRESSO (*p* = 0.207) were not significant (Supplementary Table S3), which indicated that the horizontal pleiotropy may not exist.

**TABLE 1:** Results of the MR analyses testing the causal association between benign BPH and cardiovascular disease ( $p<5\times 10^{-8}$ ).

Exposure	Outcome	MR method	Number of instruments	OR	95% CI	*p*-value
BPH	CHD	Weighted median	28	1.078	1.009, 1.151	0.026
		IVW		1.078	1.026, 1.132	0.003
		MR-Egger		1.173	0.964, 1.427	0.111
		(intercept)		−0.013	−0.042, 0.016	0.383
		MBE		1.064	0.96, 1.179	0.238
		ConMix		1.152	1.011, 1.235	0.035
		RAPS		1.080	1.032, 1.13	0.001
BPH	stroke	Weighted median	30	0.938	0.869, 1.012	0.099
		IVW		0.951	0.896, 1.01	0.102
		MR-Egger		0.936	0.742, 1.179	0.573
		(intercept)		0.003	−0.033, 0.039	0.884
		MBE		0.871	0.738, 1.027	0.101
		ConMix		0.872	0.797, 0.926	0.002
		RAPS		0.950	0.903, 0.999	0.047
BPH	Heart failure	Weighted median	29	0.993	0.958, 1.03	0.708
		IVW		0.986	0.961, 1.011	0.262
		MR-Egger		1.023	0.932, 1.122	0.633
		(intercept)		−0.006	−0.02, 0.008	0.413
		MBE		1.015	0.955, 1.079	0.639
		ConMix		0.981	0.952, 1.011	0.286
		RAPS		0.985	0.96, 1.011	0.262
BPH	Myocardial infarction	Weighted median	27	1.067	1.005, 1.132	0.034
		IVW		1.042	0.999, 1.087	0.057
		MR-Egger		1.028	0.88, 1.2	0.730
		(intercept)		0.002	−0.022, 0.027	0.855
		MBE		1.090	0.975, 1.219	0.129
		ConMix		1.107	1.022, 1.164	0.013
		RAPS		1.043	0.999, 1.089	0.058
BPH	Atrial fibrillation	Weighted median	27	1.009	0.954, 1.066	0.763
		IVW		0.990	0.942, 1.04	0.686
		MR-Egger		0.919	0.765, 1.105	0.370
		(intercept)		0.012	−0.017, 0.041	0.414
		MBE		1.042	0.956, 1.136	0.349
		ConMix		1.018	0.978, 1.07	0.412
		RAPS		0.989	0.952, 1.028	0.584
CHD	BPH	Weighted median	28	1.045	0.946, 1.154	0.390
		IVW		1.046	0.978, 1.119	0.187
		MR-Egger		1.016	0.87, 1.188	0.838
		(intercept)		0.004	−0.017, 0.025	0.684
		MBE		1.056	0.911, 1.225	0.469
		ConMix		1.057	0.918, 1.145	0.700
		RAPS		1.047	0.978, 1.122	0.188
stroke	BPH	Weighted median	16	1.092	0.936, 1.275	0.264
		IVW		1.059	0.933, 1.202	0.375
		MR-Egger		1.033	0.756, 1.413	0.838
		(intercept)		0.003	−0.033, 0.039	0.864
		MBE		0.852	0.625, 1.163	0.314
		ConMix		1.292	0.731, 1.562	0.350
		RAPS		1.063	0.951, 1.187	0.282
Heart failure	BPH	Weighted median	12	1.072	0.811.1.418	0.626
		IVW		1.128	0.885, 1.437	0.332
		MR-Egger		0.933	0.447, 1.947	0.853
		(intercept)		0.013	−0.034, 0.059	0.591
		MBE		1.041	0.693, 1.564	0.846
		ConMix		1.088	0.823, 1.69	0.494
		RAPS		1.132	0.923, 1.389	0.234
Myocardial infarction	BPH	Weighted median	22	1.010	0.912, 1.118	0.850
		IVW		1.006	0.936, 1.081	0.880
		MR-Egger		1.052	0.892, 1.24	0.547
		(intercept)		−0.007	−0.031, 0.016	0.549
		MBE		1.056	0.929, 1.202	0.405
		ConMix		1.070	0.968, 1.183	0.193
		RAPS		1.006	0.937, 1.079	0.875
Atrial fibrillation	BPH	Weighted median	47	1.033	0.955, 1.117	0.415
		IVW		0.997	0.951, 1.045	0.900
		MR-Egger		1.013	0.915, 1.122	0.797
		(intercept)		−0.002	−0.016, 0.011	0.721
		MBE		1.044	0.957, 1.138	0.333
		ConMix		1.022	0.871, 1.107	0.621
		RAPS		0.997	0.95, 1.046	0.900

The similar positive causality was found for the outcome MI, in 27 SNPs with strict thresholds (ConMix OR = 1.107, 95% CI: 1.022–1.164, *p* = 0.013; Weighted median OR = 1.067, 95% CI:1.005–1.132, *p* = 0.034) with the intercept of MR-Egger being not significant (*p* = 0.855). In addition, the results for RAPS (*p* = 0.058) and IVW (*p* = 0.057) (Table 1; Figure 1), MR-PRESSO were marginal significant (*p* = 0.063) (Supplementary Table S3).

In terms of stroke, the risk of developing stroke was found to have a significant negative causation with genetically predicted BPH (ConMix OR = 0.872, 95% CI: 0.797–0.926, *p* = 0.002; RAPS OR = 0.950, 95% CI: 0.903–0.999, *p* = 0.047) whereas the intercept for MR-Egger (*p* = 0.884) (Table 1; Figure 1) and the GlobalTest.*p*-value for MR-PRESSO (*p* = 0.067) were not yet significant (Supplementary Table S3).

**FIGURE 2:**
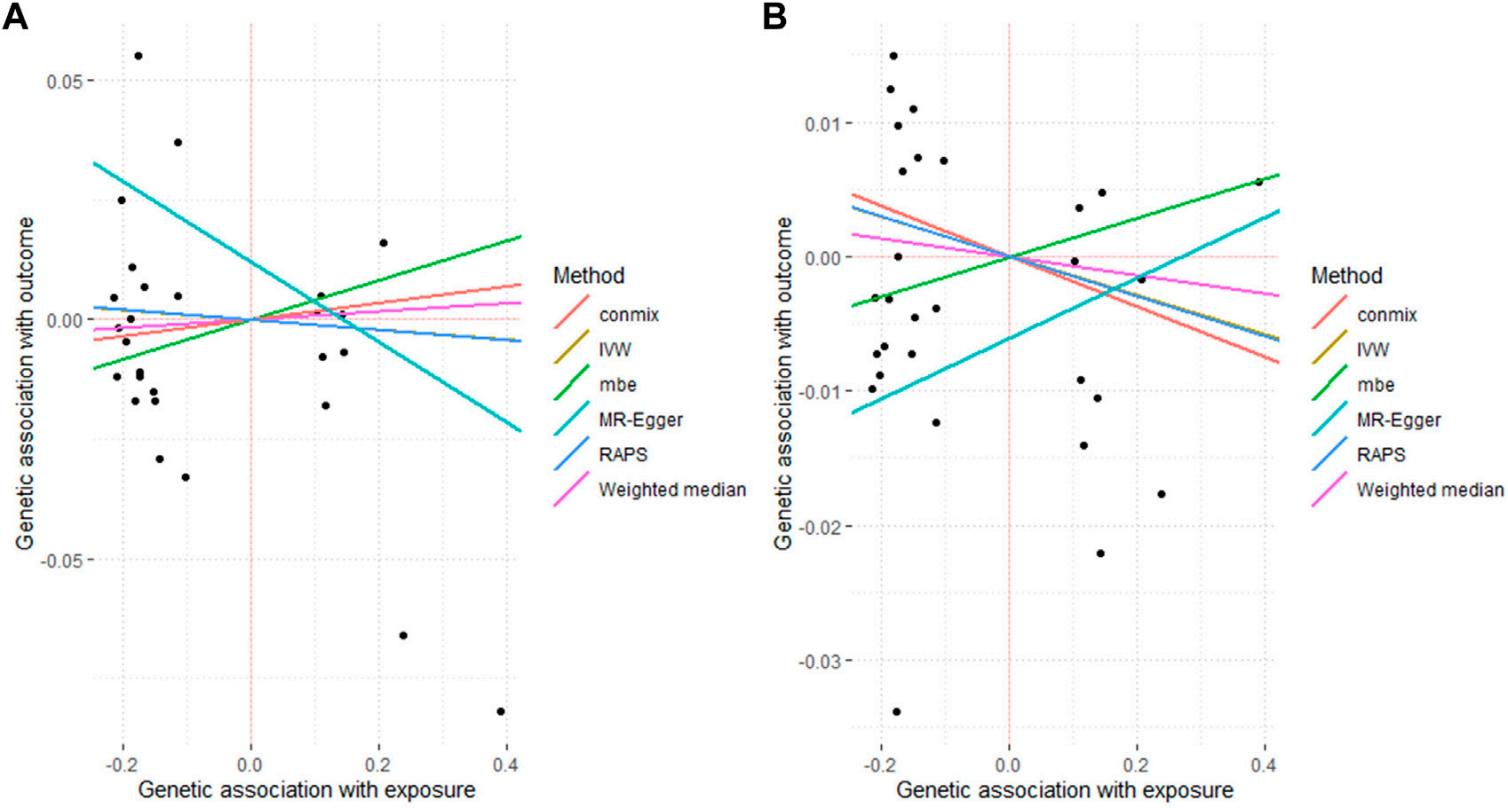
Associated plot of the main MR study investigating the causal effect of benign prostate hyperplasia on cardiovascular diseases. **(A)** outcome atrial fibrillation **(B)** outcome heart failure.

For the other two CVD traits including heart failure and AF, there was no evidence to support a causal effect of BPH at a threshold of $p<5\times 10^{-8}$ (Table 1; Figure 2; Supplementary Table S3).

### 3.2 The role of CVDs in BPH

Under the major genome-wide significance *p*-value threshold, a total of 28, 16, 12, 22 and 47 SNPs were obtained for CHD, stroke ( $p<5\times 10^{-6}$ ), heart failure, MI, and AF, respectively (Supplementary Table S2). However, none of these five cardiovascular diseases mentioned above had a significant effect on the liability of BPH (*p* > 0.05) (Table 1).

## 4 Discussion

In this study, we utilized large-scale GWAS summary statistics and a two-sample MR approach to explore the causal relationship between BPH and CVDs. Our forward analyses revealed that BPH emerged as a risk factor for CHD and MI, while demonstrating a protective effect against stroke. However, no statistically significant causal relationship was observed between BPH and AF or heart failure. Additionally, our reverse analyses failed to furnish supporting evidence for the influence of CVDs on the occurrence of BPH.

The discovery of our research that BPH served as a risk factor for CHD aligned with prior research, which identified an association between CHD and an elevated risk of moderate-to-severe BPH-related LUTS (OR = 1.68; CI: 1.08–2.61; Wald chi-square test = 5.34; *p* = 0.02) (Wong et al., 2010). Moreover, previous studies have shown that more than half of patients with BPH had erectile dysfunction (Calogero et al., 2019), significantly elevating the risk of CHD ((Dong et al., 2011)). The pathophysiology of erectile dysfunction involved oxidative stress and endothelial dysfunction induced by dyslipidemia, which is also recognized as a risk factor for CHD ((Arvanitis and Lowenstein, 2023)). Consequently, we hypothesized that dyslipidemia played an intermediary role in the causal association between BPH and CHD.

Confirming the link between BPH and MI, our study were consistent with findings from a comprehensive analysis of 702 older men aged 65–80 years (Weisman et al., 2000). This analysis revealed a significant association between BPH and an increased likelihood of experiencing MI. Existing researches consistently characterized BPH as an androgen-dependent condition (Weisman et al., 2000). Additionally, endogenous testosterone, one of the primary androgens, has been found to have a positive causal relationship with MI((Luo et al., 2019)). Therefore, our contention was that androgens played a pivotal role in mediating the impact of BPH on the occurrence of MI.

Interestingly, our discovery that BPH was protective against strokes diverged from a cross-sectional study involving 788 Chinese men, which identified an elevated risk of stroke associated with increased white matter hyperintensity in BPH patients (Yin et al., 2023). This disparity may stem from the inherent limitation of cross-sectional studies and insufficient samples, which often fails to account for causal associations. Additionally, we believe that behavioral changes associated with BPH, such as increased nocturia, may also contribute to this discrepancy. Nocturia, as a common symptom of BPH((Yoong et al., 2005)), is associated with inadequate nocturnal blood pressure reduction (Kato et al., 2024). Reduced blood pressure fluctuation could decrease vascular smooth muscle cell proliferation (Tedla et al., 2017), thereby reducing the risk of arterial stiffness and stroke (Lacolley et al., 2017).

However, our study did not identify a significant causal relationship between BPH and heart failure or AF. This finding differed from a current observational study suggesting that patients with BPH may exhibit a significantly higher risk of concomitant AF ((Hu and Lin, 2018)). The discrepancy observed could be attributed to an inadequate consideration of the causal association between BPH and AF in existing observational studies. Hence, for a more robust validation of our findings, it is crucial to utilize larger sample sizes and explore alternative methodologies, including cohort studies and genetic investigations.

Furthermore, in the reverse analysis, we discovered that these five cardiovascular diseases did not exhibit a significant causal relationship with BPH. Previous research indicated that men with metabolic diseases may undergo changes in the vascular supply and innervation of the prostate gland, ultimately resulting in an elevated cardiovascular risk and major adverse cardiovascular events (MACE) (Gacci et al., 2016). Therefore, we posited that BPH may manifest earlier than CVDs, providing an explanation for our observed results.

Our findings suggested that CVDs were severe complications of BPH, and to some extent supported that CVDs and BPH shared common risk factors in elderly men (Zhang et al., 2023). Thus, timely identification and treatment of BPH can effectively prevent the occurrence of CVDs. Moreover, previous research has demonstrated the notable impact of Pentosan polysulfate (PPS) on the proliferation of smooth muscle cells from vascular tissues and prostate smooth muscle cells, providing an efficacious treatment for both maladies (Elliot et al., 2003). The focus on drugs that can alleviate both BPH and CVDs may provide new avenues for the prevention and treatment of CVDs in patients with BPH((Milani and Djavan, 2005)). Additionally, previous research has found a strong causal relationship between lifestyle habits, particularly sleep levels, and BPH((Jia et al., 2024)). This suggests that lifestyle interventions could serve as a promising strategy for preventing CVDs in patients with BPH. By encouraging patients to adopt healthier lifestyle habits, such as improving sleep quality, the incidence and severity of cardiovascular diseases can potentially be reduced. These findings advocate for a more holistic approach in managing BPH, integrating both medical and lifestyle interventions to enhance patient outcomes and prevent cardiovascular complications.

This research has several strengths. Firstly, we utilized extensive genomic data from the UK Biobank, HERMES Consortium, and the FinnGen Genome Database, making our results more reliable. Secondly, MR analysis was employed to identify the relationship between BPH and CVDs, which could ensure the reliability of results by minimizing residual confounding to the greatest extent possible. Thirdly, we identified causal relationships between BPH and five types of CVDs, providing a new perspective for a deeper understanding of the complex interactions between BPH and CVDs.

However, there were some limitations that need to be acknowledged. Firstly, only five types of CVDs were selected in the study, and some other types of CVDs, like peripheral artery disease, may be causally related to BPH, and the correlation between them should be confirmed through subsequent studies. Besides, our study population was limited to individuals of European ancestry, and thus caution should be taken when generalizing the results to populations of other ancestries. Finally, in the correlation analysis with BPH, we encountered challenges in excluding female samples from the CVDs group. This may arise from constraints within the GWAS database, potentially introducing statistical bias.

In conclusion, our MR estimates supported an adverse effect of increased BPH on CHD and MI, while concurrently indicating a protective effect of heightened BPH on stroke in men. These established associations contributed to a more profound comprehension of the pathogenesis of both diseases, offering valuable insights for the development of more effective strategies in their prevention and treatment.

## Data Availability

The original contributions presented in the study are included in the article/Supplementary Material, further inquiries can be directed to the corresponding authors.
